# Diagnostic Value of Procalcitonin and Apo-E in Extrahepatic Biliary Atresia

**Published:** 2014-02-18

**Authors:** Mandana Rafeey, Lida Saboktakin, Jamshid Shoa Hassani, Fatemeh Farahmand, Saied Aslanabadi, Amir Ghorbani-Haghjou, Sadegh Poorebrahim

**Affiliations:** 1 Department of Pediatrics, Pediatric Health Research Center; 2 Department of Pediatrics, Tehran University of Medical Sciences, Tehran, Iran; 3 Department of Surgery; 4 Biotechnology research center, Tabriz University of Medical Sciences; 5 Danesh labarotory, Tabriz

**Keywords:** Neonatal Cholestasis, Extrahepatic Biliary Atresia, Procalcitonin, Apolipoprotein E

## Abstract

***Objective:*** Extrahepatic biliary atresia (EHBA) is one of the main causes of neonatal cholestasis. Its early diagnosis could increase the survival of the infants with early surgery. We evaluated the diagnostic accuracy of procalcitonin and apolipoprotein E (Apo-E) levels in infants with and without EHBA.

***Methods:*** This prospective study included 18 infants with EHBA and 15 infants with other causes of cholestasis. Blood samples were taken from each patient and different markers including procalcitonin and Apo-E levels were measured. ROC analysis was used to define sensitivity, specificity, positive and negative predictive value (PPV and NPV) for procalcitonin and Apo-E.

***Findings*** ***:*** There was a significantly positive correlation between Apo-E and SGOT (*r*=0.37, *P*=0.03), SGPT (*r*=0.38, *P*=0.02) and GGT (*r*=0.38, *P*=0.02), and an inverse correlation between procalcitonin and GGT (*r*=-0.45, *P*=0.01). Area under curve (AUC) for procalcitonin was 0.69 (*P*=0.05) with cut-point of 0.735 ng/ml. The sensitivity, specificity, PPV and NPV was 67%, 61%, 69% and 59%, respectively. AUC for Apo-E was 0.68 (*P*=0.06) for cut-point of 61.25 ng/ml with sensitivity, specificity, PPV and NPV of 67%, 67%, 71% and 67%, respectively.

***Conclusion:*** Both PCT and Apo-E have relatively good accuracy in diagnosing EHBA cases; we could not rely on these markers for diagnosis of EHBA, however, combinations of these biomarkers with other markers and imaging tests could improve their accuracy and may help to achieve a rapid and accurate diagnosis of EHBA.

## Introduction

Neonatal cholestasis is a common neonatal liver disease which results in diminished bile flow and excretion, and is defined serologically as prolonged conjugated hyperbilirubinemia in neonates. There are numerous causes of neonatal cholestasis^[^^1^^,^^2^^]^. Extrahepatic biliary atresia (EHBA) is the most common cause of pathologic jaundice and the most commonly recognized cause of neonatal cholestasis throughout the world and one of the most common reasons for liver transplantation in children^[^^3^^-^^6^^]^. The incidence is 1:8000 to 1:15,000 live births^[^^7^^,^^8^^]^.

 Several reports suggest that it results from prenatal injury and post-inflammatory fibrous obliteration of extra hepatic biliary tree^[^^10^^]^. Early diagnosis is essential in order to maximize the survival of the infant’s native liver^[^^9^^]^. 

 Although definitive diagnosis requires a cholangiogram, liver biopsy, and surgery, there are essential limitations such as invasiveness and need for an experienced team. Thus there is need for other surrogates for precise differentiation between BA and other neonatal cholestatic diseases^[^^9^^,^^11^^,^^12^^]^. Because of the need for early diagnosis of the EHBA, efforts are focused on biomarkers especially inflammatory markers. Since cholestasis is frequently associated with abnormalities in the lipid transport system, we have focused on plasma lipoproteins^[^^3^^,^^9^^]^. One of these markers is apolipoproteins (Apo). Apolipoprotein E (Apo-E) is an ingredient of VLDL, IDL and chylomicrons. There are increasing facts that it protects against atherogenesis through a variety of mechanisms. The Apo E gene is polymorphic and there are three common Apo-E proteins: Apo-E2, Apo-E3 and Apo-E4. The genetic variation in Apo-E has a strong effect on its anti-atherogenic properties^[^^13^^]^.

 It was shown that Apo-E is higher in serum from biliary atresia and EHBA than non-EHBA infants; such elevation is reported previously in individuals with biliary tract obstruction^[^^14^^-^^17^^]^. However, most of these findings are reported more than 20 years ago and there is a need for defining Apo-E levels in biliary atresia and EHBA. 

 Procalcitonin (PCT), the prohormone of calcitonin^[^^18^^,^^19^^]^, is a stable protein present ex vivo in plasma with a half-life of about 24–30 hr. Procalcitonin levels are shown to raise in infections and is therefore considered as a novel inflammatory marker and as an acute phase reactant biosynthesized in the liver^[^^20^^]^. PCT levels are not evaluated in neonatal cholestasis or EHBA, however, different values are reported according to the mechanism responsible for liver injury and hepatic disease^[^^21^^]^. Considering the above mentioned reports, we evaluated possible diagnostic values of Apo-E and PCT levels in EHBA.

## Subjects and Methods

Between April 2010 and 2012 all infants admitted to gastroenterology ward, Children’s Hospital, Tabriz, with jaundice at 2 weeks of age and the diagnosis of cholestasis were recruited. Patients with renal or liver failure or any disorder causing reduced production and clearance of acute phase proteins, hypoxia, shock, patients receiving antibiotics, anti-inflammatory drugs or corticosteroids before admission to the ward and patients receiving TPN were excluded. The protocol was approved by the Institutional Review Board of Tabriz University of Medical Sciences and carried out in compliance with the Helsinki Declaration. Written parental consent was obtained for each study participant during the study. 

 All neonates with cholestasis enrolled underwent physical examination, laboratory and imaging studies to define the cause for the cholestasis. Due to the results, neonates were divided into groups with extrahepatic biliary atresia (n=18) and with other causes of neonatal cholestasis (n=15). 

 Along with other laboratory evaluations, procalcitonin and Apo-E levels were also measured. Fasting blood samples were obtained via venous puncture in the first day of admission. Samples were centrifuged within 30 minutes of collection and the serum was stored at -70℃ before analysis for determination of procalcitonin and Apo-E levels.

**Determination of biomarkers**

For the determination of procalcitonin concentration, the Liaison Brahms procalcitonin assay (Diasorin S.p.A., Sallugia, Italy) was used. This assay has an improved functional assay sensitivity of 0.06 ng/ml and results range from 0.06 to 50 ng/ml. Apo-E levels were measured using Human Apolipoprotein E (Apo-E) ELISA Kit (Shanghai Crystal Day Biotech Co., Shanghai, China). This assay has an improved functional assay sensitivity of 0.23 ng/ml and results range from 0.5 to 200 ng/ml. 

**Data analysis**

Continuous data with normal distribution are given as mean±standard deviation, otherwise as median. Values are also given as the n and percent. Student t-test for testing the significance of mean for independent continuous scale data and Mann-Whitney *U *test for nonparametric data where appropriate, chi-square or Fisher exact test for testing the significance of percentages were used. 

**Table 1:** Baseline findings between patients with and without extrahepatic biliary atresia

**Variable**	**Extra hepatic biliary atresia**	**Other causes of neonatal cholestasis**	*** P*** ** value**
**Gender (male)**	9 (50%)	11 (73.3%)	0.17
**Age at biopsy/surgery**	58.72 (20.71)	52.13 (33.09)	0.49
**Age at jaundice onset (day)**	5.66 (2.66)	5.46 (1.02)	0.94
**Weight at admission (grams)**	4311.11 (1273.20)	3428.00 (1102.51)	0.04*
**Related parents**	5 (27.8%)	10 (66.7%)	0.02*
**Acholic stool**	17 (94.4%)	11 (73.3%)	0.15
**Positive cord sign**	4 (22.2%)	2 (13.3%)	0.66
**Concomitant anomalies**	1 (5.6%)	5 (33.3%)	0.07

* *P* is two-sided significant

ROC analysis was conducted to calculate sensitivity and specificity of Apo-E and procalcitonin. *P*<0.05 was considered as significant. All statistical analyses were performed with SPSS version 17.0 (SPSS, Chicago, IL, USA).

## Findings

In this study, 33 neonates with mean age of 55.72±26.80 days referring with jaundice were evaluated. HIDA scan was performed in 31 patients and results were indicative of extrahepatic biliary atresia (EHBA) in 26 (83.9%), suspected atresia in 1 (3.2%) and no atresia in 4 (12.9%). Among 33 patients, only 22 underwent diagnostic laparatomy and cholangiography during laparatomy, this showed atresia in 13 (59.1%). Twenty-six patients who had liver biopsy and pathologic findings showed EHBA in 16 (61.6%), neonatal hepatitis in 9 (34.6%) and paucity in 1 (3.8%). The missing cases are due to disagreement of parents for further imaging or invasive evaluations. 

 Final diagnosis was made according to imaging studies, laboratory and laparatomy findings; 18 patients were defined to have EHBA and 15 patients had other causes including neonatal hepatitis in 12, paucity in 1, α1-Antitrypsin deficiency in 1 and inborn error of metabolism (IEM) in 1 patient. 

 Baseline findings between patients with and without EHBA are shown in Table 1. Patients with extrahepatic biliary atresia had significantly higher weight at admission and rate of consanguinity in parents were low.

Table 2 demonstrates laboratory findings between patients with and without extrahepatic biliary atresia. 

**Table 2:** Laboratory findings between patients with and without extrahepatic biliary atresia

**Variable**	**Extra hepatic ** **biliary atresia**	**Other causes of ** **neonatal cholestasis**	***P*** **value**
**Total bilirubin (mg/dl)**	12.29 (4.97)	12.54 (4.18)	0.89
**Direct bilirubin (mg/dl)**	6.71 (2.70)	7.80 (3.03)	0.28
**SGOT (U/l)**	274.88 (48.95)	337.26 (136.29)	0.64
**SGPT (U/l)**	163.83 (29.26)	187.06 (67.00)	0.73
**ALP (U/l)**	1784.66 (683.73)	1220.26 (723.00)	0.02*
**Gamma-glutamyl transpeptidase (U/l)**	583.88 (445.40)	242.65 (190.72)	0.01*
**PT**	14.18 (4.18)	12.95 (1.74)	0.29
**PTT**	40.33 (13.41)	35.13 (7.81)	0.19
**Albumin (g/dl)**	3.64 (0.50)	3.92 (0.47)	0.11
**Apo-E (** **ng/ml)**	133.19 (90.48)	81.02 (72.48)	0.08
**Procalcitonin (** **ng/ml)**	1.37 (2.91)	1.47 (1.33)	0.90

* *P.* value is two-sided significant.

**Fig. 1:**
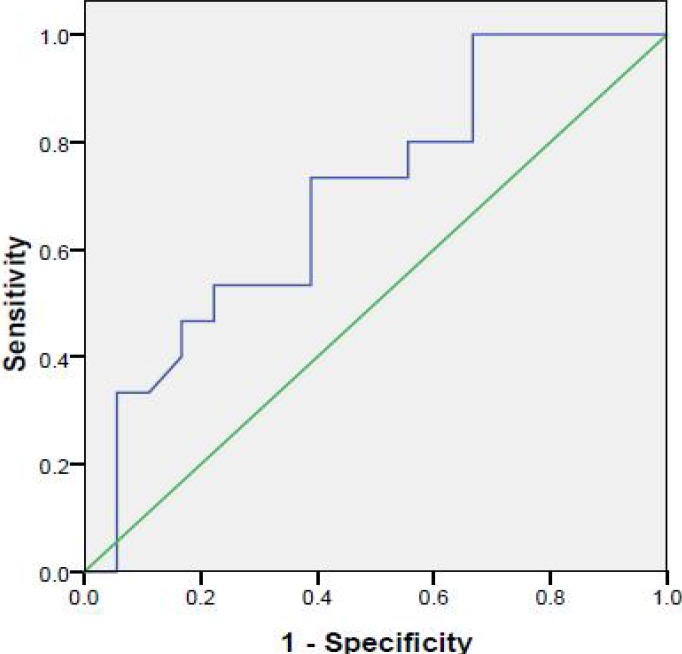
ROC curve for procalcitonin in differentiating cases with and without EHBA

Besides total and direct bilirubin, all other laboratory findings were higher in patients with extrahepatic atresia; however, only Gamma-glutamyl transpeptidase and alkaline phosphatase (ALP) had statistically significant difference.

 We evaluated the correlation between Apo-E and procalcitonin with laboratory findings and found significant positive correlation between Apo-E and SGOT (*r*=0.37, *P*=0.03), SGPT (*r*=0.38, *P*=0.02) and Gamma-glutamyl transpeptidase (*r*=0.38, *P*=0.02), as well as inverse correlation between procalcitonin and Gamma GT (*r*=-0.45, *P*=0.01). The inverse correlation between Apo-E and procalcitonin was not significant (*r*=-0.03, *P*=0.84). We also found positive correlation between Apo-E levels and weight at admission (*r*=0.35, *P*=0.04) and between procalcitonin levels and age at jaundice onset (*r*=0.34, *P*=0.04). 

 Procalcitonin levels in extrahepatic biliary atresia group were between 0.28 and 1.2 in 17 cases and 13 in a female infant which had cholangitis and died after 69 days. The range of procalcitonin in the other group was between 0.47 and 4.52. Procalcitonin was 4.52 in a female neonate with severe sepsis. Due to the lower levels of procalcitonin in extrahepatic biliary atresia group, ROC curve analysis was used to define cut-point of procalcitonin in diagnosing cases with and without extrahepatic biliary atresia (Fig. 1). AUC was 0.69 (*P*=0.05) with cut-point of 0.735 ng/ml. The sensitivity, specificity, positive and negative predictive value (PPV and NPV) of procalcitonin in differentiating cases with and without extrahepatic biliary atresia with cut-point of 0.735 ng/ml was 67%, 61%, 69% and 59%, respectively.

**Fig. 2:**
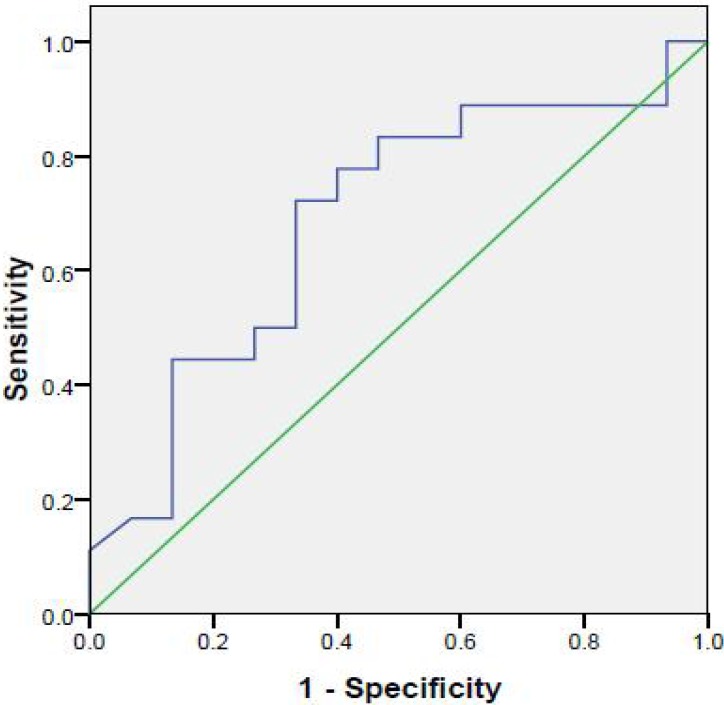
ROC curve for Apo-E in differentiating cases with and without EHBA

 We also used ROC curve to define probable cut-points for Apo-E in diagnosing extrahepatic biliary atresia from other causes of jaundice (Fig. 2). Area under curve (AUC) was 0.68 (*P*=0.06). Although not significant, the evaluated cut-point was 61.25 ng/ml. The calculated sensitivity, specificity, PPV and NPV was 67%, 67%, 71% and 67%, respect-tively.

## Discussion

While idiopathic neonatal hepatitis and EHBA are the major causes of neonatal cholestasis, EHBA must be managed operatively as soon as possible; the preferred treatment of less common metabolic and other causes is medical^[^^22^^-^^24^^]^. 

 Distinguishing between different underlying etiologies of neonatal cholestasis is of great importance because the plan of treatment could literally vary^[^^23^^]^. 

 Late referral of infants with neonatal cholestasis is considered to be one of the main problems in dealing with these patients; especially in developing countries^[^^9^^,^^24^^]^.

Similarly, most EHBA cases in our study more likely have been referred late, but the difference was not significant. 

 As the previous study by Nemati and colleagues, in our study it was also shown that HIDA scan although is very sensitive in diagnosis of EHBA, its positive predictive value was low^[^^25^^]^. 

 More than 80% of cases with EHBA, who undergo Kasai portoenterostomy before 60th day of life become jaundice-free, as compared to 20-35% that were operated later. In the infants with successful biliary drainage, a 15-year survival of 87% has been shown^[^^26^^]^. Among 18 neonates with EHBA, nine underwent Kasai portoenterostomy, one died due to severe cholangitis and for the other eight liver transplantation was recommended because of liver cirrhosis due to late referral and late diagnosis. 

 Since immediate portoentrostomy may prevent further potentially fatal consequence of EHBA, it is very critical to differentiate EHBA from other causes of cholestatic jaundice^[^^23^^,^^27^^,^^28^^]^. Many modalities and markers are proposed for this purpose. We evaluated different laboratory findings and observed that although most markers were higher in EHBA, only Gamma-glutamyl transpeptidase and ALP had statistically significant difference. Similarly, Chu and colleagues^[^^29^^]^ showed that SGOT, SGPT, ALP and Gamma-glutamyl transpeptidase were higher in biliary atresia; however, in this study total and conjugated bilirubin was also higher. 

 Among these modalities, due to previous reports we suspected that Apo-E and procalcitonin levels could distinguish between EHBA and other causes. We found positive correlation between Apo-E and Gamma-glutamyl transpeptidase and inverse correlation between procalcitonin and Gamma-glutamyl transpeptidase. It is known that procalcitonin levels increase by age, as we observed a positive correlation between procalcitonin levels and age at jaundice onset. 

 Over the past two decades, the body of literature on the clinical usefulness of procalcitonin in adults has grown rapidly. However, the use of PCT in pediatric populations is less evaluated and is mostly studied for severe infection and sepsis diagnosis^[^^30^^]^. PCT has been proposed as a marker of bacterial sepsis in critically ill patients. PCT is a precursor of calcitonin and a 116 amino acids protein^[^^18^^,^^19^^]^. In healthy persons, PCT levels are barely detectable^[^^18^^,^^19^^]^. It is believed that hepatic cells are potential sources for PCT^[^^31^^]^. Due to the potential role of hepatic cells in producing PCT, it is possible that PCT increase in different liver diseases could differentiate these diseases especially biliary tract involvement. It was previously reported that acute alcoholic hepatitis and acute viral hepatitis on cirrhotic background without proven bacterial infection induce mild elevation of serum PCT levels^[^^32^^,^^33^^]^. However, in another study, there was no statistical correlation between PCT serum levels and the presence of biliary obstruction on ERCP findings^[^^34^^]^. 

 As mentioned before, there are few studies evaluating PCT levels in diseases other than sepsis in infants. Korczowski^21^ evaluated serum procalcitonin in children with hepatic disease. They observed that procalcitonin level was low in viral infection and toxic liver injury, and was moderately elevated in 50% of children with an autoimmune process. These bring up the possible role for procalcitonin in distinguishing between different liver diseases including neonatal cholestatic disease. In this study, sensitivity, specificity, PPV and NPV of procalcitonin in differentiating EHBA from other cases was 67%, 61%, 69% and 59%, respectively. Our findings are indicative of the role of procalcitonin, but the low sensitivity and specificity as well as low PPV and NPV question its utility. 

 The possible role of various apolipoproteins is mentioned previously. Unlike PCT, Apo-E has been previously studied in various liver diseases and in neonatal cholestasis^[^^14^^-^^17^^,^^35^^]^ indicating that Apo-E levels increase in biliary atresia, especially EHBA. Similar to these findings, in our study Apo-E levels were significantly higher in EHBA patients in comparison to other causes of neonatal cholestasis. The sensitivity, specificity, PPV and NPV of Apo-E in distinguishing between these was 67%, 67%, 71% and 67%, respectively. Apo-E with slightly better PPV and NPV seems to be a better marker than PCT in diagnosing EHBA cases. However, both markers have low sensitivity and specificity which limits their diagnostic use.

## Conclusion

Our findings show that both PCT and Apo-E have relative accuracy in diagnosing EHBA cases; we could not rely on these markers for diagnosis of EHBA, however, combinations of these biomarkers with other markers and imaging tests could improve their accuracy and may help for rapid and accurate diagnosis of EHBA. However, further studies should be performed to define the exact level of significance and to confirm these findings.
